# Increased *H19*/miR-675 Expression in Adult T-Cell Leukemia Is Associated with a Unique Notch Signature Pathway

**DOI:** 10.3390/ijms25105130

**Published:** 2024-05-08

**Authors:** Marcia Bellon, Christophe Nicot

**Affiliations:** Department of Pathology and Laboratory Medicine, University of Kansas Medical Center, Kansas City, KS 66160, USA; mbellon@kumc.edu

**Keywords:** ATL, HTLV, leukemia, lymphoma, Notch, *H19*, miR-675, JAG, Myc, Hes, Hey, FBXW7

## Abstract

The Notch pathway is a key cancer driver and is important in tumor progression. Early research suggested that Notch activity was highly dependent on the expression of the intracellular cleaved domain of Notch-1 (NICD). However, recent insights into Notch signaling reveal the presence of Notch pathway signatures, which may vary depending on different cancer types and tumor microenvironments. Herein, we perform a comprehensive investigation of the Notch signaling pathway in adult T-cell leukemia (ATL) primary patient samples. Using gene arrays, we demonstrate that the Notch pathway is constitutively activated in ATL patient samples. Furthermore, the activation of Notch in ATL cells remains elevated irrespective of the presence of activating mutations in Notch itself or its repressor, FBXW7, and that ATL cells are dependent upon Notch-1 expression for proliferation and survival. We demonstrate that ATL cells exhibit the expression of pivotal Notch-related genes, including *notch-1*, *hes1*, *c-myc*, *H19*, and *hes4*, thereby defining a critical Notch signature associated with ATL disease. Finally, we demonstrate that lncRNA *H19* is highly expressed in ATL patient samples and ATL cells and contributes to Notch signaling activation. Collectively, our results shed further light on the Notch pathway in ATL leukemia and reveal new therapeutic approaches to inhibit Notch activation in ATL cells.

## 1. Introduction

Adult T-cell leukemia (ATL) is characterized by clonal or oligoclonal expansion of T-cells infected by the human T-cell leukemia virus-I (HTLV-I). ATL has one of the most dismal prognoses of all leukemias, is invariably fatal, and has poor survival rates that vary with disease classification. Four-year survival rates of acute and lymphoma-type ATL, which represent the more advanced, less favorable disease states, vary from 5.0% to 5.7%, respectively [1]. Recent longitudinal studies indicate that favorable survival times are only achieved in indolent ATL, made up of smoldering or favorable-chronic type ATLs [2]. The progression of ATL in HTLV-I-infected individuals occurs after a long latency, and it is estimated that around 5–10% of HTLV-I individuals will develop ATL in their lifetime. Though the exact number of HTLV-I infected individuals is likely under-estimated, it is believed that in ubiquitous, hot-spot regions, 1–2% of persons are infected, with some foci reaching 20–40% of the population over the age of 55 [3]. HTLV-I infection appears to initiate the pathway toward ATL development; however, it is unknown whether pre-existing conditions or genetic defects exist before HTLV-I infection that make a host cell vulnerable to ATL development [4]. Once infected, the expression of HTLV-I proteins, specifically the viral Tax protein, instigates genome and genetic instability, allowing for T-cell transformation that culminates in the development of ATL [5]. This exact process is still largely unknown, including what specific genetic and cellular changes are required to promote ATL.

Expression of the HTLV-I Tax protein results in a random mutagenesis model in T-cells, leading to the accumulation of genetic defects. Tax disrupts the cell cycle and DNA repair pathways, creating an environment conducive to genetic mistakes [6]. Large-scale genetic screens have identified several genes that are frequently mutated in primary ATL patients [7]. Studies have found *prkcb* (protein kinase C-β), *plcg1* (phospholipase C γ-1), *tp53* (tumor protein p53), *tbl1xr1* (transducin β like 1 X-linked receptor 1), *gata3* (GATA binding protein 3), *ccr4* (C-C motif chemokine receptor 4), *irf4* (interferon regulatory factor 4), *card11* (caspase recruitment domain family member 11), *notch1* (Notch receptor 1), *km2td* (MLL2; lysine methyltransferase 2D), *stat3* (signal transducer and activator of transcription 3), *rhoA* (Rho homolog family member A), *ccr7* (C-C motif chemokine receptor 7), and *tet2* (TET methylcytosine dioxygenase 2) are among the top genes mutated in ATL patients that alter ATL leukemogenesis and pathogenesis of disease [7,8,9,10,11,12]. We have found that 30% of ATL patients harbor activating mutations in Notch-1 [13]. The Notch pathway was first linked to T-cell acute lymphoblastic leukemia (T-ALL), where it was found that over 60% of all T-ALL patients harbored mutations, predominantly in the heterodimerization (HD) domain of Notch [14]. In contrast, ATL mutations were found in the PEST (proline–glutamate–serine–threonine) domain of Notch, the majority leading to decreased degradation by the F-box and WD repeat domain containing 7 (FBXW7/CDC4), ubiquitin ligase complex [13]. We also found that 25% of ATL patients harbor FBXW7 mutations, which prevented interaction and, hence, degradation of Notch or other targets by the FBXW7 complex [15].

Notch signaling is critical in ATL disease, given the mutually exclusive mutations in Notch-1 and FBXW7. In support of this, treatment of ATL cells with γ-secretase (GSI) inhibitors, which prevent the generation of NICD, causes a loss in proliferation, cell cycle disruption, and decreased tumor growth in an ex vivo ATL mouse model [13]. Enhanced tumor growth is also seen in an ex vivo mouse model harboring ATL cells with FBXW7 mutations unable to degrade Notch [15]. We have also demonstrated that elevated expression of *notch* ligands, including *jag1*, is involved in Notch activation in ATL cells. Indeed, treatment of ATL cells with a monoclonal antibody targeting JAG1 prevented Notch signaling and cellular migration [16]. Considering the existence of genetic mutations affecting different elements of the Notch pathway, the vulnerability of ATL cells to disruptions in Notch and FBXW7, and the upregulation of genes implicated in the Notch pathway, our focus was directed towards determining the presence of a Notch activity signature in ATL cells. The goals of this study were to determine the following: 1. if some ATL cells are more dependent upon Notch activity than others, 2. if ATL cells harboring wild-type Notch/FBXW7 still have an active Notch pathway, and 3. which transcriptional targets define Notch activity in ATL cells, i.e., a Notch signature for ATL disease. To accomplish this, we carried out Notch signature gene arrays in ATL cells. We found that Notch-1 is the dominant receptor in ATL cells, which are susceptible to proliferation losses in its absence. We also find ATL patients express heightened levels of Notch binding partners, including fringe genes, *dtx1* (Deltex E3 ubiquitin ligase 1), *rbpjk* (recombination signal binding protein for immunoglobulin kappa J region), and *mamls* (mastermind-like transcriptional coactivator). We establish a Notch signature of direct Notch target genes in ATL that includes *hes-1*, *hes-4*, *hey-1*, *hey-2*, and *c-myc*. We also discovered over-expression of the lncRNA (long non-coding RNA), *H19*, in primary ATL patients and demonstrated that *H19* is a component of a Notch activity signature and plays a role in regulating Notch signaling in ATL cells.

## 2. Results

### 2.1. Expression of Notch Receptors, Ligands, and Binding Partners in ATL Cells

**Figure 1:**
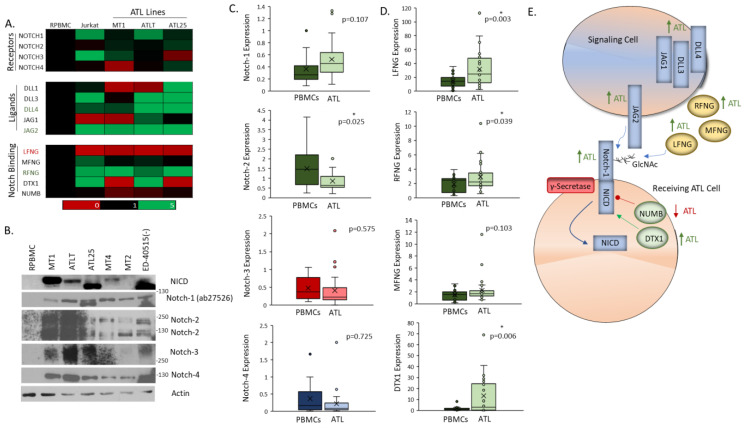
Notch-1 and binding partners are over-expressed in adult T-cell leukemia (ATL) cells. (**A**) Gene Array analysis of Notch receptors, ligands, and binding partners in control, healthy peripheral blood mononuclear cells (PBMCs), Jurkat cells, and three ATL lines (MT1, ATLT, and ATL25). Expression is normalized to control PBMCs and is color-coded to represent expression levels: green (up to 5-fold) is elevated fold-change compared to PBMCs. Red is repressed fold-change compared to PBMCs. Black is equal to control PBMCs (set at 1). (**B**) Western blot analysis of Notch receptors, Notch-1, -2, -3, and -4, including Notch intracellular domain (NICD), expression in ATL cell lines (MT1, ATLT, ATL25, and ED-40515(−)) and human T-cell leukemia virus-I (HTLV-I) infected cells (MT4 and MT2). Non-infected PBMCs served as control. Actin served as a loading control. Protein weight in kilodaltons (kDa) is shown. Notch-1 is detected with the ab27536 antibody, and NICD is detected with the NICD-Val1744 antibody. (**C**,**D**) Whisker plots of real-time PCR gene expression analysis of Notch receptors (*notch* 1–4) (**C**) in a cohort of healthy PBMCs (n = 17), primary ATL patient samples (n = 23), and Notch binding partners (*lfng, mfng, rfng*, and *dtx1*) (**D**) in a cohort of healthy PBMCs (n = 18–20) and primary ATL patient samples (n = 22–28); *p*-values are indicated. The colors of whisker blots indicated relative levels of expression (green: highly expressed, red: low expression, and blue: medium expression). (**E**) Graphical representation of Notch signaling in ATL cells. Green arrows represent elevated expression of genes, while red arrows represent decreased expression of genes.

To determine if ATL cells harbor a Notch pathway signature, we performed a Notch gene expression array using three ATL-derived cell lines, MT1, ATLT, and ATL25. MT1 has an activating PEST domain mutation in Notch, which produces the active, cleaved Notch intracellular domain (NICD). ATLT and ATL25 also express activated NICD (see Figure 1A,C). We compared ATL cells to normal peripheral blood mononuclear cells (PBMCs) and the T-cell acute lymphoblastic (ALL) cell line, Jurkat. The Jurkat cell line was used for comparison since most T-ALL patients have activating mutations in Notch [14]. Jurkat cells express high levels of NICD due to an internal tandem mutation in exon 28 of Notch and likely loss of PTEN and mutations in FBXW7 [17,18]. PBMCs are a standard control for ATL cells, as CD4 and CD8 T-cells, B-cells, myeloid, and plasmacytoid dendritic cells are known to be infected with HTLV-1 [19,20,21,22,23]. Notch activity is mediated by four Notch receptors (Notch 1–4), Notch ligands, Notch binding partners, and Notch co-transcriptional activators [24]. Our gene array demonstrated variable levels of Notch receptor expression across ATL lines and Jurkat, which was confirmed by real-time RT-PCR on a larger panel of ATL lines (Figure 1A and Supplementary Material Figure S1B). Overall, we found that ATL cells consistently expressed high levels of *notch-1*, while *notch-3* and *-4* were expressed lower or undetectable in some lines. These results were consistent at the protein level, where we found ATL cells expressed higher levels of Notch-1 and NICD but lower levels of Notch-2, -3, and -4 (Figure 1B). To determine if these observations translated to primary ATL patient samples, we examined Notch receptor gene levels by RT-PCR in a cohort of normal, non-infected PBMCS vs. primary ATL patients’ cells. Although *notch-1* gene expression was the only receptor appreciably elevated compared to normal cells, *notch-2* gene expression was highly expressed in both ATL and PBMCs (Figure 1C). However, the *notch-3* gene exhibited the lowest expression among the receptors in ATL cells and primary patient samples. Poor Notch-3 expression could be due to differences in antibodies or small sample size, as we did not analyze cleaved Notch-3, which has been reported to be expressed in a minor portion of primary T-ALL tumors (2 of 40) [25]. In this case, it could be surmised that ATL cells more generally resemble B-ALL lines, in which Notch-3 is found hypermethylated [26,27]. Notably, a portion of T-ALL patients have been found to harbor activating Notch-3 mutations [25,27]. An integrated analysis of ATL patient samples did find one ATL patient with a stop-gain mutation in Notch-3 (Q2395*), but to our knowledge, this is the only Notch-3 mutation reported for ATL [7]. This is in contrast to Notch-1, in which a large portion of ATL patients exhibit Notch mutations [7,13]. Notch receptors are bound by families of the jagged ligands (JAG1 and JAG2) and delta-like ligands (DLL1, DLL3, and DLL4). We have previously shown that JAG1 and DLL4 are elevated in Tax-expressing cells, and our array confirms this observation, as both ATLT and ATL25 (which express Tax protein) expressed higher amounts of these ligands compared to MT1, which do not express Tax protein (Figure 1A) [16]. In general, ATL and ALL cells expressed higher gene levels of *jag2*, *dll3*, and *dll4* compared to normal PBMCs.

When examining Notch binding proteins, several trends appeared. A distinct difference in the expression of the glycosyltransferase 31 gene family among different cell lines was found. This family consists of three glycosyltransferases that transfer a GlcNAc to *O*-fucose residues onto Notch: MFNG (maniac fringe), LFNG (lunatic fringe), and RFNG (radical fringe). *O*-fucose residues strongly alter Notch receptor affinity for its ligand and subsequent Notch signaling [28]. We found that ATL (and ALL) cells tended to over-express *rfng*, while *lfng* was repressed (Figure 1A). The *mfng* gene also appeared to be repressed in ATL cells compared to PBMCS and Jurkat cells. However, examination of Notch binding partners in primary ATL patients showed that all glycosyltransferases were over-expressed in ATL patients compared to normal PBMCs (Figure 1D), which may lead to optimal Notch signaling [29]. This is due to the fact that the presence or absence of various fringe proteins dictates how Notch ligands can induce Notch-1 signaling and that the effect of LFNG is dominant over other fringes that may be present [30,31]. The fact that *lfng* mRNA is significantly over-expressed in ATL patient samples may suggest that LFNG plays a dominant role in dictating signaling through various JAG and DLL ligands in ATL patients. While Notch signaling is enhanced in ATL patients through elevated fringe expression, Numb (NUMB endocytic adaptor protein), a negative regulator of Notch, was expressed at lower gene levels in all tested cells. *Dtx1*, an E3 ubiquitin ligase that has regulatory roles in Notch activity, was elevated in ALL cells compared to most ATL. ATLT expressed high levels of *dtx1*, which was analogous to primary ATL patient samples (Figure 1A,D). This suggests a model of Notch signaling in ATL cells, whereby Notch-1 is the primary driver of signaling through JAG-1, -2, DLL-2, -3, and -4, whose activity is enhanced in the presence of fringe proteins, especially LFNG (Figure 1E). Notch-1 activity is likely maintained in ATL patients through lower expression of Numb and higher DTX1 expression.

### 2.2. ATL Cells Are Dependent on Notch-1 Activity to Maintain Proliferation

**Figure 2:**
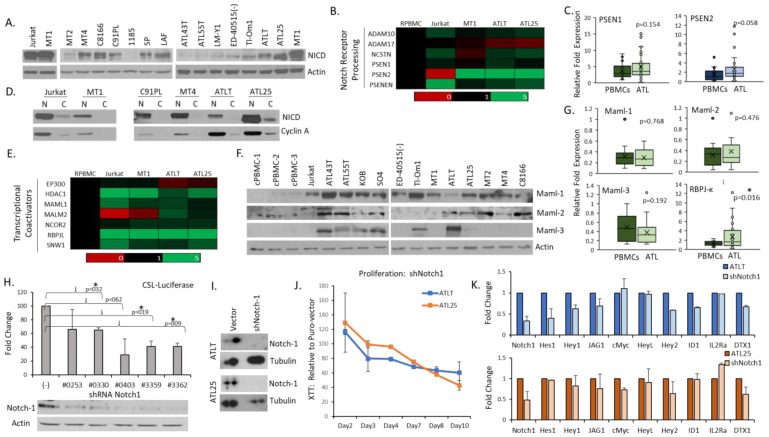
Adult T-cell leukemia (ATL) cells are dependent upon Notch-1 for Growth. (**A**) Western blot analysis of active Notch (NICD-Val1744) expression in ATL cell lines (MT1, ATLT, ATL25, ED-40515(−), Tl-Om1, ATL43T, ATL55T, and LM-Y1) and human T-cell leukemia virus-I (HTLV-I) infected cells (MT4, MT2, C8166, C91PL, 1185, SP, and LAF). Non-HTLV-I infected cells served as controls. Actin served as a loading control. (**B**) Gene Array analysis of Notch receptor processing genes in control, healthy peripheral blood mononuclear cells (PBMCs), Jurkat cell line, and three ATL lines (MT1, ATLT, and ATL25). Expression is normalized to control PBMCs and is color-coded to represent expression levels, as stated in Figure 1. (**C**) Whisker plots of real-time PCR analysis of *presenilin* genes in a cohort of healthy PBMCs (n = 18) and primary ATL patient samples (n = 28). The colors of whisker blots indicated relative levels of expression (green: highly expressed, red: low expression, and blue: medium expression). (**D**) Nuclear (N) and cytoplasmic (C) Western blot analysis of NICD in ATL and HTLV cells. Jurkat cells served as a control, with nuclear Cyclin A verifying the purity of cellular fractionation. (**E**) Gene array analysis of Notch transcriptional coactivators, as described in (**B**). (**F**) Western blot analysis of Malm proteins in ATL cell lines (MT1, ATLT, ATL25, Tl-Om1, ATL43T, ATL55T, KOB, SO4 and ED-40515(−)) and HTLV-I-infected cells (MT4, C8166, and MT2). Three non-infected cycling PBMCs (cPBMCs) and Jurkat cells served as control. Actin served as a loading control. Protein weight in kilodaltons (kDa) is shown. (**G**) Whisker plots of real-time PCR analysis of *maml* and *rbpjk* genes in a cohort of healthy PBMCs (n = 18–20) and primary ATL patient samples (n = 22–28). *p*-values are indicated. * = significance less than 0.05. The green color of whisker blots indicates a high level of expression of all genes. (**H**) CSL-Luciferase in transfected 293T cells. Five Notch shRNA were transfected, along with the CSL-luciferase vector. Control cells were transfected with empty vectors. Experiments were performed at least twice, and fold change was determined compared to the empty vector. *p*-values are indicated. * = significance less than 0.05. Corresponding protein levels of Notch-1 (ab27526) and Actin are shown for each shRNA. (**I**) Western blot analysis of HTLV and ATL cells infected with shRNA to Notch-1. Actin served as a control. (**J**) Proliferation of ATLT and ATL25 cells infected with empty vector or Notch-1 shRNAs was performed using XTT assays. Assays were performed at least twice, and XTT assays were performed in triplicate. Values represent the mean measurements compared to control cells on Days 2, 3, 4, 7, 8, and 10 days. (**K**) Real-time PCR analysis of Notch-associated genes following knock-down of Notch-1 by lentiviral infection of shRNA. Cells were infected at least twice, and the mean value was graphed as a fold change compared to vector-only cells. GAPDH served as an internal control.

Our data indicate that Notch-1 was preferentially expressed in ATL cells and patient samples compared to normal PBMCs. Notch-1 expression, however, is not an indicator of Notch activity, as Notch-1 undergoes a series of proteolytic cleavage events to produce the active form, NICD. We expanded upon our previous findings [13] and found NICD expression in all ATL cells examined, though the level and size of NICD varied greatly between cell types (Figure 2A). Our results indicated that Notch-1 activity is a general characteristic of ATL cells and that IL-2 (interleukin-2) dependent, immortalized ATL cells expressed lower levels of NICD compared to HTLV-I transformed or ATL, IL-2 independent cells. This suggests that IL-2 signaling may parallel or compensate for a fully active Notch pathway and warrants further studies. Acquisition of an active Notch pathway may be an important step toward ATL cellular transformation. The initial cleavage of NICD is carried out by ADAM metalloproteases, whereas the second cleavage is catalyzed by the γ-secretase complex, which consists of presenilin proteins [32,33]. Our gene expression data indicated that ATL cells express slightly elevated levels of *adam-10* and presenilin-1 (*psen1*) and even higher levels of presenilin-2 (*psen2*) (Figure 2B). ATL cells likely use PSEN2 as part of the active γ-secretase complex required to cleave Notch to active NICD [34]. Indeed, the *psen2* gene was over-expressed in primary ATL patient samples and ATL cells, along with *psen1* (Figure 2C). After γ-secretase cleavage, NICD is translocated to the nucleus to bind DNA. We found nuclear NICD in ATL cells, demonstrating proper localization of an active Notch (Figure 2D). Once NICD translocates to the nucleus, it binds indirectly to a Notch transcriptional complex consisting of the DNA binding protein RBPJ/CSL and one of the mastermind-like proteins (Mamls). Mamls are transcriptional coactivators for Notch receptors that potentiate the activity of NICD [35]. Mamls show little differences between paralogs Maml-1, -2, and -3 for Notch/RBP-J binding. Studies have shown that Jurkat cells primarily express Maml1, likely due to Maml1’s role in lymphocyte development (Supplementary Material Figure S1A) [36]. This contrasts with ATL cells, which showed strong gene expression of all three *mamls*, which aligned with protein expression data (Figure 2E,F). There was a wide variation in the expression of the *maml* genes between different ALL and ATL cell lines (Supplementary Material Figure S1C). However, examination of primary ATL patient samples indicated strong expression of *mamls* in primary patient samples (Figure 2G). Our array also highlighted the high gene expression of *rbp-J* in all leukemic cell lines (Figure 2E and Supplementary Material Figure S2A). When RBP-J is bound to NICD, it produces a highly active transcriptional complex by recruiting chromatin remodeling machinery to the DNA. We found that the gene expression of *rbp-j* (RBPJκ, also known as RBP-J or CSL) was significantly increased in ATL patients compared to normal PBMCs (Figure 2G). Studies have shown that RBPjκ has a strong role in canonical Notch signaling [37]. In the absence of NICD, RBPjκ is a repressor of Notch transcriptional targets, such as Hes-1 [38]; however, in the presence of NICD, as is the case in ATL cells, a switch occurs to activate Notch transcriptional targets. In support, we have already shown that ATL cells are sensitive to the knockdown of Maml [13]. The fact that NICD is constitutively expressed at some level in ATL cells demonstrates the breakdown in Notch repressor signals, contributing to ATL persistence.

Our data suggests that the over-expression of Notch-1, RBPjκ, and Mamls, control ATL growth and the regulation of Notch transcriptional targets and that ATL cells are addicted to Notch activation [39]. To further verify this and to validate our expression data on Notch-1, we used individual Notch-1 shRNAs to infect ATL cells. We transfected five Notch-1 shRNA vectors into 293T cells and measured their ability to inhibit Notch activity, as measured by CSL-luciferase activities. We found that all five of the Notch-1 shRNAs were capable of repressing Notch activity (Figure 2H). Individual Notch-1 shRNA could also knock down CSL-luciferase activity in the presence of active NICD. We then infected ATL lines with several lentiviruses expressing Notch-1 shRNAs. We confirmed the knock-down of Notch-1 by Western blot (Figure 2I) and gene expression (Figure 2K) in ATL cells. We then infected ATL lines with shNotch-1 and examined proliferation levels over ten days. We found that ATL cells, with high NICD expression, were dependent on Notch-1 activity, as cells progressively died following long-term expression of Notch-1 shRNA (Figure 2J). Importantly, the loss of Notch-1 led to the inhibition of known Notch target genes, such as *hes-1*, *hey-1* (Hes-related family BHLH transcription factor with YRPW motif 1), *hey-2*, *jag1*, *id1* (inhibitor of DNA binding 1), and *dtx1* (Figure 2K). As expected, one of the Notch target genes, *IL2Ralpha* (CD25; IL-2 receptor subunit α), was less affected, as this gene is known to be regulated by Notch-independent pathways in ATL cells [40,41,42].

### 2.3. High Notch Activity in Cells Exposes a Notch Pathway Signature in ATL Cells

**Figure 3:**
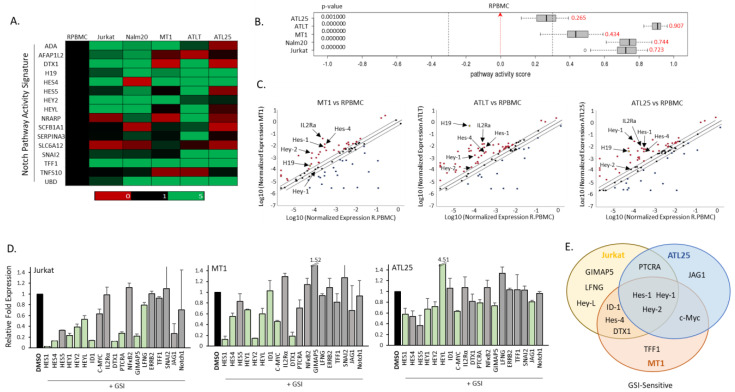
Adult T-cell leukemia (ATL) cells have a high notch pathway activity score. (**A**) Gene array analysis of Notch pathway activity signature genes in control, healthy peripheral blood mononuclear cells (PBMCs), two ATL lines (Jurkat, T-ALL and Nalm20, B-ALL), and three ATL lines (MT1, ATLT, and ATL25). Expression is normalized to control PBMCs and is color-coded to represent expression levels, as stated in Figure 1. (**B**) Notch pathway activity score generated from array data and Qiagen pathway analysis. PBMCs served as a control, with a score of 0.0. ATL and ALL cell scores are in red and represent an elevation compared to PBMCs. *p*-values are indicated. (**C**) Graphs representing the Log10 normalized gene expression of Notch pathway genes of ATL cells (MT1, ATLT, and ALT25) compared to the Log10 normalized gene expression in PBMCs. Graphs are generated using Qiagen software. (**D**) Real-time PCR analysis of Notch targets in Jurkat, MT1, and ATL25. GAPDH served as a control. Results are from at least two distinct cDNA preparations, from at least two different experiments and real-time PCR runs. Cells were treated with 1 µM GSI for 48 h. Fold change is compared to cells treated with DMSO control. Significant decreases are indicated in pale green with *p*-values of at least > 0.05. (**E**) Venn diagram representing common GSI-sensitive targets among Jurkat (yellow), MT1 (red), and ATL25 (blue).

Studies suggest that predicting Notch activity cannot be based solely on the presence of Notch-activating mutations—either in Notch or its regulator, FBXW7 [43]. In fact, NICD expression is high in most ATL cells, even in the absence of known mutations (Figure 2A). Indeed, *Hes-1*, considered the top prototypical canonical Notch target gene, is highly expressed in most ATL cells, including *hey-1* (Supplementary Material Figure S2B) [44]. Of the cells tested, however, only MT1 and Jurkat harbor known mutations in Notch, and neither cell line harbored mutations in FBXW7 (of note, not all cell lines were tested) [18]. The expression of key conserved Notch genes can produce a quantitative score that allows prediction of whether cells would have high Notch activity through NICD expression in the absence of known driver mutations [43]. Researchers have procured a Notch activity signature score using the mean expression derived from an examination of Notch transcriptome targets, such as an 18 gene score for human cancers, a 20 gene score for breast cancer, and a 15 gene score for glioblastoma multiforme [43,45,46]. These activity scores are developed to determine the activation of the Notch pathway and allow researchers to determine Notch sensitivity within a cell without performing extensive genomic or proteomic work. We performed a Notch pathway activity signature array developed by Qiagen that selects 16 experimentally derived Notch signature biomarker genes, which, along with classification algorithms, acquire a quantitative analysis to determine a Notch activity score for ATL and ALL cells. These genes included: *cd44*, *dtx1*, *ephb3* (ephrin type-B receptor 3), *hes-1*, *hes-4*, *hes-5*, *hes-7*, *hey-1*, *hey-2*, *heyL*, *myc* (MYC proto-oncogene), *nfkb2* (p100/p52: nuclear factor kappa B subunit 2), *nox1* (NADPH oxidase 1), *nrarp* (NOTCH regulated ankyrin repeat protein), *pbx1* (PBL; Pre-B-cell leukemia transcription factor 1), *pin1* (protein interacting with never In mitosis A1), *plxnd1* (plexin D1), and *sox9* (SRY-Box transcription factor 9). We compared three ATL and two ALL cell lines to normal, healthy PBMCs (Figure 3A). Using PBMCs as a base value, we determined a Notch activity score for each cell line. We found all ATL and ALL cell lines had higher Notch activity compared to PBMCs (Figure 3B). Surprisingly, ATLT cells had higher Notch activity than Jurkat and MT1, cell lines with known Notch activating mutations.

Examination of the gene array in ATL cells vs. normal PBMCs demonstrated several Notch genes were over-expressed in ATL cells (Figure 3C). This was also true of ALL cell lines (Supplementary Material Figure S2A). We then verified which genes were direct Notch transcriptional targets, i.e., GSI-sensitive in ATL cells. We decided to treat ATL cells with a GSI inhibitor, as this would reveal genes that required a functional, active Notch protein for transcription. We examined the expression of a variety of Notch transcriptional targets in ATL cells, including promoter proximal Notch binding genes (i.e., *hes-1*, *hey-1*, *heyL,* and *hes-4*) and distal enhancer Notch binding genes (i.e., *c-myc* and *dtx1*) following NICD inhibition [36,47,48]. We found common GSI-sensitive, NICD-dependent targets among Jurkat and ATL lines: *hes-1*, *hey-1*, and *hey-2* (Figure 3D,E). We also found *c-myc*, *IDI*, *ptcra*, *dtx1*, *hey-1*, *gimap-5* (GTPase, IMAP family member 5), and *jag1* were more prone to be NICD-dependent in T-cell lines (Figure 3D). Analysis of *hes-1*, *hey-1*, and *c-myc* gene expression in a panel of ATL cell lines verified that these genes were highly expressed in ATL cells (Supplementary Material Figure S2B). Surprisingly, *c-myc* was down-regulated in both ATL lines following GSI treatment (Figure 3D,E), suggesting c-Myc is highly dependent upon NICD in ATL cells. Our previous results demonstrated high gene expression of *c-myc* in ATL primary patient samples, which was not dependent upon the expression of viral Tax or HBZ [49]. We have also shown that c-Myc protein is degraded by FBXW7 in ATL cells. This, combined with our new data, suggests that the down-regulation of FBXW7 allows the continued expression of NICD, which regulates c-Myc in ATL cells. We also found that genes involved in cell signaling pathways that have a critical role in ATL transformation, such as NF-κB (*nkfb2*) and IL2 signaling (*IL2Rα/CD25*), were constitutively active but insensitive to NICD loss (Figure 3D). This is not surprising, as a role for HBZ, Tax, and NF-κB signaling in elevation of the IL2Rα gene, and Tax in regulating NFKB2 gene expression in ATL cells, has already been demonstrated [40,50].

**Figure 4:**
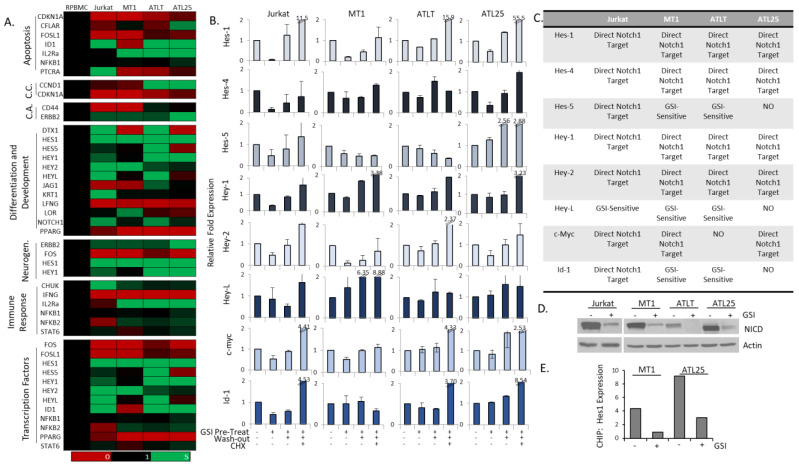
Determination of direct Notch transcriptional targets in adult T-cell leukemia (ATL) cells. (**A**) Gene array analysis of Notch targets involved in apoptosis, cell cycle (C.C.), cell activation (C.A.), differentiation and development, neurogenesis, immune response, and transcription factors in control, healthy peripheral blood mononuclear cells (PBMCs), Jurkat cells, and three ATL lines (MT1, ATLT, and ATL25). Expression is normalized to control PBMCs and is color-coded to represent expression levels, as detailed in Figure 1. (**B**) GSI-washout experiments were performed in Jurkat and ATL cells (MT1, ATLT, and ATL25). Cells were treated with 1 μM GSI for 72 h, followed by washout with or without 40 μg/μL CHX. Experiments were conducted at least twice, and RT-PCR was performed on Notch target genes. Cells treated with DMSO served as controls. (**C**) Table summarizing gene expression from GSI-washout experiments. Direct Notch targets were defined as loss of gene expression following GSI treatment, which was rescued upon washout and CHX treatment. (**D**) Western blot analysis of Jurkat and ATL cell lines treated with 1 µM GSI for 72 h. Actin served as a control. NICD is detected with the NICD-Val1744 antibody. (**E**) In vivo ChIP of the Hes-1 promoter following GSI treatment in MT1 and ATL25 cells. Results represent fold enrichment compared to DMSO-treated controls.

Examination of normalized gene expression of ATL cells vs. PBMCs found that a wide array of Notch-related genes were elevated in ATL cells (Figure 3C). These included genes in the analysis of the Notch Activity score: *hes-4*, *hey-2*, *hey-L*, and lncRNA *H19*, but also genes *hes-1*, *hey-1*, and *IL2Rα*. The gene array found varying levels of expression of Notch transcriptional targets involved in apoptosis, cell cycle, cell activation, differentiation and development, neurogenesis, the immune response, and transcription factors (Figure 4A). In general, Notch-related apoptosis genes were down-regulated (*cdkn1A* (cyclin-dependent kinase inhibitor 1), *cflar* (CASP8 and FADD-like apoptosis regular), *fos* (AP-1: Fos proto-oncogene), *fosl1* (Fos-like 1), and *ptcra* (Pre T-cell antigen receptor alpha)), while Notch-related transcription factors were elevated (*hes-1, hes-5*, *hey-1*, *hey-2*, *hey-L*, *id1*, and *nfkb2*).

Identification of direct Notch transcriptional targets is best determined using GSI-washout experiments. This takes advantage of the fact that GSI treatment is reversible, and the addition of cycloheximide (CHX) prevents secondary translational effects [51,52]. Parallel treatment with CHX determines if de novo protein synthesis is required, indicating direct vs. indirect targets. As previously shown, *hes-1* is a direct NICD target in Jurkat cells, as *hes-1* expression was elevated in CHX-treated washout cells (Figure 4B). This also proved to be the case in ATL cells, MT1, ATLT, and ATL25. Our results also demonstrated that *hey-1*, *hes-4*, *hey-1*, *hey-2*, and *c-myc* were also direct NICD targets in ATL cells (Figure 4B). *Id1* was a direct target in Jurkat cells but not in ATL cells. Notch activation provided an advantage in survival in an ID1-expressing T-ALL transgenic mouse model [53]. A role for ID1 in the bone marrow microenvironment has been described for AML; however, ID1 has not been studied in ATL [54]. The results of GSI-washout experiments are summarized in Figure 4C. *Hey-L* and *hes-5* showed sensitivity to GSI but were mostly indirect NICD targets in cells (Figure 4B,C). We also validated GSI treatment, as GSI-treated cells prevented the generation of the active NICD fragment (Figure 4D), and in vivo ChIP analysis demonstrated a reduction in Notch-1 bound to the Hes-1 promoter in ATL cells treated with GSI (Figure 4E).

### 2.4. Primary ATL Patient Cells Express High Levels of the H19/miR-675 Gene Locus

**Figure 5:**
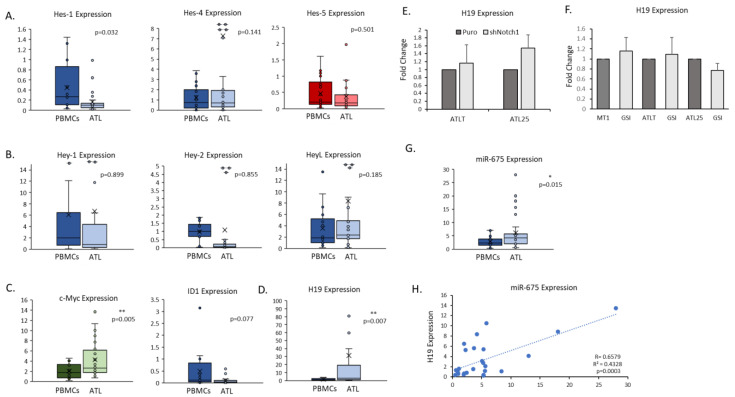
Primary adult T-cell leukemia (ATL) patients over-express Notch targets, including lncRNA *H19*. (**A**–**D**) Whisker plots of real-time PCR analysis of Hes transcription factors (*hes-1*, *-4*, and *-5*) (**A**), Hey transcription factors (*hey-1*, *-2*, -L) (**B**) and transcription factors (*c-myc* and *id1*) (**C**) and lncRNA (*H19*) (**D**) in a cohort of healthy PBMCs (n = 16–18), and primary ATL patient samples (n = 28–29). *p*-values are indicated. The colors of whisker blots indicated the level of expression (Green: highly expressed, Red: low expression, and Blue: Medium expression). (**E**) Real-time PCR analysis of *H19* lncRNA expression following knock-down of Notch-1 by lentiviral infection of Notch-1 shRNA. Cells were infected at least twice, and the mean value was graphed as a fold change compared to vector-only cells. GAPDH served as an internal control. (**F**) Real-time PCR analysis of *H19* lncRNA in MT1, ATLT, and ATL25 cells treated with GSI. GAPDH served as a control. Results are from at least two distinct cDNA preparations, from at least two different experiments and real-time PCR runs. Cells were treated with 1 µM GSI for 48 h. Fold change is compared to cells treated with DMSO control. (**G**) Whisker plots of mir-675 expression, as described in (**A**). (**H**) Graph plotting miR-675 expression vs. *H19* expression in corresponding primary ATL patient samples. The correlation coefficient and *p*-values are listed. * means significance less than 0.05.

After confirming Notch direct and indirect targets in ATL cells, we next examined the expression of Notch-related genes in primary ATL patient samples. We found high expression of *hes-1* in ATL patient samples (Figure 5A). Though mean *hes-1* expression was not over-expressed in ATL patients compared to PBMCs, we found individual ATL patients had very high expression. We have shown that this higher *hes-1* gene expression correlated with stronger NICD levels in ATL patients [13]. Lower *hes-1* levels in ATL patients may be skewed compared to PBMCs. Monocytes, which make up 10–20% of PBMCs, and naïve B-cells, which make up 5–10% of lymphocytes in PBMCs, are known to have higher levels of *hes-1*; whereas CD4 and CD8 T-cells do not express high levels of *hes-1* (Human Protein Atlas) [55,56]. The Hes family is composed of seven members, but only Hes-2, Hes-3, and Hes-6 appear to be Notch independent, whereas all three Hey family members (Hey-1, Hey-2, and Hey-L) are strongly induced by Notch [57]. Both families can bind E-box elements found in gene promoters; however, Hes proteins can also bind N-box elements and contain a WRPW domain that can bind distinct co-repressors. Examination of other Hes family members indicated high expression of *hes-4* in ATL patient samples (Figure 5A). We also found high expression of *hey* family members in ATL patient samples, especially *hey-L* (Figure 5B). Surprisingly, we found *hey-2* to be down-regulated in ATL patient samples. Hey-2 acts as a strong co-repressor of TGF-β signaling by binding to Smad-3/-4 [58]. The TGF-β pathway is complex in HTLV-I, with viral proteins contributing to the activation and repression of various TFG-β components. It could be that Hey-L plays a part in TGF-β signaling in ATL patients. In agreement with past studies, we found over-expression of *c-myc* gene expression in ATL patients compared to PBMCs (Figure 5C) [49]. We also found down-regulation of *id1* in ATL patient cells (Figure 5C). The fact that *id1* was not impacted by NICD loss and is down-regulated in ATL patients suggests that ID1 may have tumor suppressor functions in ATL cells that are currently undefined. However, like *hes-1*, PBMCs have high levels of *id1*, likely due to the higher expression of *id1* in monocytes, basophils, neutrophils, and myeloid dendritic cells (Human Protein Atlas) [56]. Additional investigations into the role of ID1 in ATL disease are warranted. One surprising result that came from the Notch array was the high expression of the Notch-related lncRNA *H19* in ATL cells (Figure 3D). *H19* was one of the most highly over-expressed genes in ATL cells compared to PBMCs (Figure 3A). We, therefore, examined *H19* expression in primary ATL patients compared to PBMCs. Like ATL cells, we found over-expression of *H19* in primary ATL patient samples (Figure 5D). *H19* is an imprinted lncRNA on chromosome 11p15.5 that is maternally expressed [59]. A previous report found that ATL patient cells display loss of imprinting (LOH) at the *H19* allele, leading to a gain of expression from the lost parental allele [60]. Additional reports have suggested that Notch receptors could positively regulate *H19* expression [61]. To determine if Notch signaling also contributed to elevated *H19* expression, we infected ATL cells with Notch-1 shRNA. We did not find a loss of *H19* expression when Notch-1 was knocked down (Figure 5E). Furthermore, inhibition of NICD with GSI did not prevent *H19* gene expression (Figure 5F). These results show that *H19* is not a direct Notch-1 target in ATL cells. Though we did see a slight increase in *H19* expression following shNotch1, the result was not statistically significant. Studies have shown that a portion of H19’s oncogenic effects is due to the expression of microRNA, miR-675, located in the first exon of *H19* [62]. We, therefore, examined miR-675 expression in ATL patient samples and found miR-675 was also over-expressed compared to normal PBMCs (Figure 5G). We also found the *H19* expression correlated with miR-675 expression in ATL patient samples, suggesting both non-coding RNAs are dually regulated (Figure 5H).

### 2.5. H19/miR-675 Regulates Notch Signaling

**Figure 6:**
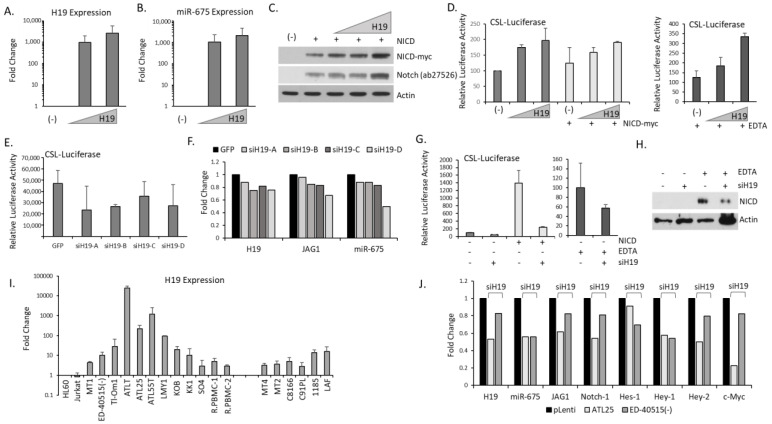
The *H19*/miR-675 locus regulates Notch activity. (**A**,**B**) Real-time PCR analysis of *H19* (**A**) and miR-675 (**B**) expression in 293T transfected with increasing amounts of H19-pCDNA. GAPDH served as an internal control. Results are from at least two distinct cDNA preparations, from at least two different transfection and real-time PCR runs. (**C**) Western blot analysis of myc-tagged NICD and Notch-1 in 293T transfected with myc-NICD and increasing H19-pCDNA. NICD is detected with myc antibody, and Notch-1 is detected with ab27526 antibody. Actin served as an internal control. Cells transfected with the vector served as a negative control. (**D**) CSL-luciferase activity in 293T cells transfected with increasing amounts of H19-pCDNA with or without myc-NICD and with and without exposure to 20 min of EDTA. Transfections were performed at least twice, and mean values were graphed in comparison to the empty vector, which was set at 100. (**E**) CSL-luciferase activity in 293T cells transfected with individual lentiviral siRNA to *H19*. Transfections were performed at least twice, and mean values were graphed in comparison to the empty vector, which was set at 100. (**F**) RT-PCR expression of *H19*, miR-675, and *jag1* in 293T transfected with individual siH19. Values were graphed as a fold change compared to vector-only cells. GAPDH served as an internal control. (**G**) CSL-luciferase activity in 293T cells transfected with all four lentiviral siRNA to *H19* with or without myc-tagged NICD and with or without 20 min exposure to EDTA. Transfections were performed at least twice, and mean values were graphed in comparison to the empty vector, which was set at 100. (**H**) Western blot analysis of endogenous NICD expression (Val1744) in 293T transfected with *H19* siRNA. Actin served as a control. (**I**) RT-PCR analysis of *H19* expression in ATL cell lines (MT1, ATLT, ATL25, ED-40515(−), Tl-Om1, ATL43T, ATL55T, KOB, KK1, SO4, and LM-Y1) and HTLV-I infected cells (MT4, MT2, C8166, C91PL, 1185, and LAF). Non-HTLV-I infected cells, HL60 and Jurkat, served as controls, along with two resting PBMCs (rPBMCs). GAPDH served as a control. Results are from at least two distinct cDNA preparations and real-time PCR runs. (**J**) RT-PCR analysis of ATL25 and ED-40515(−) transduced with *H19* siRNA or empty vector. Values were graphed as fold change compared to empty vectors. GAPDH served as an internal control.

We next examined whether the H19/miR-675 gene locus influences overall Notch activity. We used an H19-expressing plasmid cloned into the pCDNA vector, which efficiently expressed *H19* after transfection into 293T cells (Figure 6A). We also found similar levels of miR-675 expressed from the *H19* plasmid, confirming that *H19*/miR-675 are expressed from the same locus (Figure 6B). Co-transfection of *H19* with a myc-tagged NICD plasmid led to elevated NICD levels, as demonstrated by increased protein expression of myc-NICD and Notch (Figure 6C). We then examined whether *H19*/miR-675 could affect the Notch CSL reporter vector. Our experiments demonstrated that *H19* led to a dose-dependent increase in CSL activity in the presence or absence of NICD (Figure 6D). Basal CSL activity was elevated more with *H19* than with over-expression of NICD. Additionally, we treated cells with the chelating reagent EDTA (ethylenediaminetetraacetic acid), which leads to ligand-independent NICD production. We found that *H19*/miR-675 also increased Notch activity in the presence of endogenous NICD (Figure 6D). Importantly, four *H19* siRNAs individually cloned into a pLenti-siRNA-GFP vector and transfected along with a CSL-luciferase led to significant decreases in CSL activity (Figure 6E). Transfection of individual siH19 led to decreases in *H19* and miR-675 expression (Figure 6F). Studies have shown that in mesenchymal stem cells, *H19* can regulate a group of miRNAs regulating Notch activity through ligands and receptors [63]. Previous reports have also identified a WNT/β-catenin-*H19*-JAG1 pathway in colorectal cancer [64]. We found that the knock-down of *H19*/miR-675 was sufficient to cause decreases in *jag1* expression with all siH19 tested (Figure 6F). siH19 was also able to reduce basal, and NICD-myc transfected CSL activity (Figure 6G). In agreement with *H19* over-expression data, we found siH19 also reduced EDTA-induced CSL-activity and endogenous NICD protein levels (Figure 6G,H). Collectively, these data demonstrate that while *H19* is not a direct NICD target in ATL cells, *H19* positively regulates Notch and NICD activity in cells. Finally, we examined *H19* expression in a panel of ATL cell lines. *H19* expression was highly expressed in ATL cell lines, with ATL-derived patient cells generally expressing very high levels of *H19*, compared to HTLV-I-infected cells in vitro and normal PBMCs (Figure 6I). In addition, ATL and HTLV-I cell lines had higher expression of *H19* compared to non-HTLV cells, Jurkat, and HL60. We then infected ATL lines with siH19. We found decreased expression of the *H19*/miR-675 locus after siH19 transduction in two ATL lines (Figure 6J). Similar to 293T cells, we also demonstrate that siH19 could reduce *jag1* and *notch-1* expression, following *H19*/miR-675 knock-down in two ATL lines. We then examined key Notch targets and found reduced expression of *hes-1, hey-1, hey-2*, and *c-myc* following *siH19* transduction. This demonstrates that knock-down of the *H19*/miR-675 locus in ATL cells reduces Notch activity.

## 3. Discussion

The Notch pathway is essential in cancer development and progression [65]. Previous work by our laboratory found mutations in Notch and FBXW7, along with decreased ATL cell proliferation in the presence of Notch inhibitors, JAG1 monoclonal therapy, and knock-down of Maml [13,15,16]. In this study, we deepen our understanding of Notch signaling in ATL cancer cells. We confirm the persistence of essential elements of Notch canonical signaling in ATL cells, which actively contribute to ATL cell proliferation. Specifically, we find that ATL cells are defined by elevated Notch-1 and Notch-dependent *c-myc* expression. We also demonstrate that *hes-1*, *hes-4*, *hey-1*, and *hey-2* are direct Notch targets in ATL cells.

A limitation of studying the global analysis of Notch family gene expression in ATL patient samples compared to control PBMCs is inherent to ATL diseases. ATL patients can present with smoldering, chronic, or acute types of disease with great variations in the amount of circulating tumor cells [66]. In fact, as little as 5% of circulating abnormal T-cells are required to diagnose disease [1]. In addition, this is complicated by the fact that HTLV-1 infects multiple lineages in vivo, including CD4 T-cells, CD8 T-cells, B-cells, and monocyte dendritic cells [20,21,22,23]. In our studies, ATL-extracted RNA samples represent PBMCs from individuals with ATL disease, and these samples were compared to RNA extracted from PBMCs from normal healthy individuals. While some genes such as *notch-2*, *hes*, and *hey* are highly expressed in monocytes compared to CD4 T-cells (Database of Immune Cell Expression (https://dice-database.org/landing; accessed on 10 January 2024), there is no evidence in the published literature that the relative percentage of circulating monocytes is affected in ATL patients. It is also important to consider that the expression of these genes is likely to be affected by soluble factors secreted by HTLV-1-transformed cells rather than the absolute number.

While enhanced activity of NICD is vital for the up-regulation of these genes, other cellular pathways important in ATL cells may also contribute to their expression. Studies suggest that over-expression of NICD could impact transcription driven by factors such as SMADs, HIF-1α, and NF-κB [38]. TNF-α can induce the expression of Hes-1 and Hey-1 in a Notch-dependent pathway that requires IKK2 (inhibitor of nuclear factor kappa B kinase subunit beta) [67]. IKK2 leads to enhanced Histone H3 phosphorylation and downstream activation of *hes-1* transcription. IKK1/IKK2 have known roles in NF-κB activation in HTLV-I-infected cells [68]. TGFβ/Smad can also interact with NICD, enhancing *hes-1* transcription [69]. The HTLV-I protein, HBZ, increases TGFB/Smad signaling, and therefore, it may also contribute to enhanced expression of Notch transcriptional targets [70]. Recently, studies have shown that super-enhancers are capable of regulating a wide swath of genes. These regions contain elevated levels of transcription factors and display characteristics of active chromatin: monomethylation of lysine at position 4 of histone 3 and acetylation of lysine at position 27 of histone 3 [71]. Super-enhancers have been found in genes involved in T-cell activation (*IL2RA/CD25*, *CD30,* and *FYN*) and known cancer genes (*TP73*, *CCR4*, *TIAM2*, *NFATC1*, *NFATC2*, and *PIK3R1*) in ATL cells [72]. It would be informative in future studies to focus on whether genes in the Notch pathway also contain super-enhancers, such as *Hes/Hey* and *c-myc*. This has been demonstrated for T-ALL, in which a Notch-MYC (N-Me) super-enhancer has a critical role in Notch targeted expression of Myc expression [73].

Research has also shown that Notch binding partners can associate with proteins independent of Notch. We found high expression of *rpb-J* and Maml proteins in ATL patients and ATL cells. These Notch coactivators may be complexed with non-Notch partners in ATL cells, such as β-catenin, MEF2C, and p53, and require further study [74,75,76]. The enhanced expression of Notch genes may also impact cross-talk between other cellular signaling pathways important for ATL growth and survival. We and others have demonstrated that ATL patients are characterized by high mutational rates in STAT3 and are sensitive to JAK/STAT3 inhibition [7,9,49,77]. Interestingly, studies have shown that Hes/Hey proteins can partner with JAK/STAT proteins [78]. Hes-1 and Hes-5 can associate with JAK2 and STAT3, leading to STAT3 phosphorylation and nuclear localization. Hey-1 and Hey-2 can also bind STAT3 and enhance STAT3 transcriptional activity [57,78]. Therefore, our studies suggest a strong connection between elevated Notch signaling and constitutive STAT3 activation in ATL cells.

For the first time, we report that the *H19*/miR-675 locus is highly up-regulated in ATL cells and primary ATL patient samples. We think this is due to the loss of imprinting at *H19*, which has been found in a portion of ATL patient cells [60]. *H19* was increased in AML patients, independent of miR-675, and has been found to play a role in leukemogenesis [79]. T-ALL and B-ALL patients express elevated *H19* through enhanced c-Myc expression [80]. *H19* was required for efficient induction of Bcr-Abl-induced tumorigenesis [81]. While *H19* expression is increased in numerous leukemias and has both tumor-inducing and tumor maintenance roles, *H19* has also been shown to promote and maintain the cancer stem cell phenotype [82]. Furthermore, *H19* can promote chemo-resistance through enhanced oncogene expression, epigenetic modulation, enhanced cell proliferation, and promoting metastasis. Previously, we demonstrated the existence of cancer stem cells derived from the side population of Tax transgenic mice [83]. These cells were capable of self-renewal and had lower levels of Notch1 activation. STAT3 and β-catenin/Wnt3 expression were also lower, suggesting a link with Notch/Hes/Hey/STAT3 in ATL cells. While *H19*/miR-675 expression was not directly examined in ATL cancer stem cells, it will be interesting to see if this locus plays a role in maintaining cancer stem cells in a Notch-independent manner. It may be that *H19*/miR-675 up-regulates Notch signaling in a non-side population, normal ATL cells to maintain ATL proliferation, but has a very different role in ATL cancer stem cells.

In conclusion, we have identified a specific Notch signature in ATL cells. We find that ATL cells are dependent upon Notch-1 and hes-1 and that *hey-1*, *hey-2*, and *c-myc* are important, direct Notch targets in ATL cells. We also find strong expression of the *H19*/miR-675 locus in ATL cells and ATL primary patient samples, suggesting that *H19*/miR-675 may contribute to enhanced Notch activity in ATL cells by regulating *notch-1* and *jag1* expression and represent a novel target for ATL.

## 4. Material and Methods

### 4.1. Cell Culture and Patient Samples

ATL cell lines (MT1, ATLT, ATL25, ED-40515(−), Tl-Om1, MT2, MT4, C8166, C91PL), ALL cell lines (Jurkat and Nalm-20), and HL60 were maintained in RPMI 1640 media (Invitrogen) with 10% fetal bovine serum (FBS) (Bio-Techne). ATL cell lines (ATL43T, ATL55T, LM-Y1, KK1, KOB, SO4, 1185, LAF) were supplemented with 50 U/mL IL-2 and 20% FBS. Primary ATL patient samples have been used in previous studies [4,9]. ATL patient samples were obtained after informed consent. Patient samples were in agreement with the regulations for the protection of human subjects and internal institutional review board approval. Control samples consisted of peripheral blood mononuclear cells (PBMCs) from healthy, non-infected (HTLV-I–negative) individuals. The number of PBMCs and ATL patient samples used in real-time gene expression analysis is indicated by Supplementary Figure legends and Supplementary Tables. All samples were taken from whole blood, and PBMCs were isolated using Ficoll density gradients.

### 4.2. RNA, Real-Time PCR, and Gene Array Analysis

RNA from whole cell lysates of cell lines and ATL primary patient samples were obtained by using Trizol (Invitrogen), followed by chloroform/isopropanol extraction. DNA contamination was removed with Turbo DNaseI (Invitrogen, Carlsbad, CA, USA) using the manufacturer’s protocols. cDNA was generated using random primers with the High-Capacity RNA-to-cDNA MultiScribe reverse transcriptase kit (Applied Biosystems, Foster City, CA, USA). cDNA was used in real-time PCR reactions with SYBR green (ABClonal, Woburn, MA, USA) or iTaq (Bio-Rad, Hercules, CA, USA) solutions using the StepOnePlus RT-PCR machine (Applied Biosystems, Foster City, CA, USA). GAPDH served as an internal control. Primers are listed in Supplementary Material Figure S4. RT^2^ Profiler PCR Array Human Notch Signaling Pathway Plus (Qiagen, Hilden, Germany) was used for gene arrays. This array encompasses pre-derived Notch-related genes deposited onto a standard 96-well real-time PCR plate. Gene array analysis was performed using Qiagen pathway analysis. (Qiagen, Hilden, Germany). Values and ClusterGram analysis of genes are presented in Supplementary Material Figure S3. Select array genes were verified by designing unique gene primers (Supplementary Material Figure S4), and new primers were used in additional real-time PCR applications on larger sample sets.

### 4.3. Statistics

For ATL patient gene analysis, *p*-values were calculated using a 2-tailed distribution Student’s *t*-test, using unequal variance. Correlation values were determined using Pearson’s correlation coefficient (R) using the Social Science Statistics (https://www.socscistatistics.com/). Correlation graphs and values were determined using Excel statists on Microsoft Windows 10. For experiments using 293T transfection or GSI-treated samples, *p*-values were calculated using a 2-tailed distribution Student’s *t*-test, using paired analysis. *p*-values less than 0.05 were considered significant and are noted as *p*-value: >0.05 “*”, *p*-value: >0.005 “**”, and *p*-value > 0.001 “***”. All fold changes, standard deviations, *p*-values, and *t*-test values are reported in Supplementary Material Figure S5. For real-time PCR data for patient samples, the average PBMC and ATL patient fold changes, number of patient samples tested for each gene, and statistical *p*-values are provided in Supplementary Material Figure S5.

### 4.4. Gene Knock-Down

The Notch-1 shRNAs are cloned into the pLKO.1 vector and were purchased from Sigma Aldrich. Five of the shRNAs target the Notch-1 CDS coding sequence, TRCN0000320403, TRCN0000003362, TRCN0000350253, TRCN0000350253, and TRCN0000350330, and one, TRCN000000359, targets the Notch-1 3’UTR. The *H19* siRNAs were cloned into the piLenti-siRNA-GFP vector and were purchased from Applied Biological Materials. To verify knock-down, individual Notch shRNA and siH19 were separately transfected into 293T cells for luciferase, gene, and/or protein expression. For knock-down into ATL cells, individual Notch shRNA and H19 siRNAs were transfected into 293T cells using the Calcium Phosphate Kit (Invitrogen, Carlsbad, CA, USA), along with the pDLN and VSV-G lentiviral packaging plasmids. Notch-1 shRNA lentivirus or H19 siRNA lentivirus were collected from 293T supernatant following several days post-transfection. Supernatants were spun at high speed to pellet residual 293T cells and passed through 0.22 μM filters to purify the virus. ATL cells were then infected by spinoculation with filtered lentiviral supernatants and polybrene (Sigma-Aldrich, St. Louis, MI, USA). Spinoculation was repeated every 24 h with individual Notch and *H19* virus for several days to efficiently infect ATL cells. Cells were grown in normal RPMI following transduction. Virus generated with empty vector served as a negative control.

### 4.5. Cellular Transfection and Luciferase

For *H19* expression studies, 293T cells were transfected with increasing doses of *H19*-pCDNA vector (pcDNa3.1(+)A009-H19; #122473 Addgene) by Polyfect transfection reagent (Qiagen, Hilden, Germany). For experiments with Notch, a myc-tagged Notch intracellular domain (NICD-myc) plasmid was co-transfected with *H19*. For luciferase assays, 293T cells were transfected with the Notch/CSL-firefly luciferase (CSL-Luciferase) construct by Polyfect (Qiagen, Hilden, Germany). Then, 48 h later, cells were lysed in a passive lysis buffer and assayed using the Dual Luciferase-Reporter kit (Promega, Madison, WI, USA). Fold change was determined compared to control (empty vector) transfected cells. EDTA (ethylenediaminetetraacetic acid) was added to induce ligand-independent NICD production in 293T cells.

### 4.6. Protein Analysis

For protein analysis, whole cell lysates from ATL and ALL cell lines were lysed in radioimmunoprecipitation assay buffer (RIPA). Protein lysates were quantified using BSA Bradford (BioRad) standard curves. An equal amount of protein lysate was loaded onto gels according to Actin quantification. Standard SDS/PAGE electrophoresis was performed, and blots were probed with the following antibodies: cleaved Notch1 (Val1744) (Cell Signaling), Notch-1 (ab27526) (Abcam), Notch-1 (ab27526) (Abcam), Notch-2 (C-2) (Santa-Cruz, Dallas, TX, USA), Notch-3 (A-6), Notch-4 (A-6) (Santa-Cruz, Dallas, TX, USA), Maml-1 through-3 (One World Labs), myc(9E10), and Actin (Santa-Cruz, Dallas, TX, USA). For nuclear and cytoplasmic protein expression, cells were lysed in hypertonic and hypotonic buffers, respectively, according to previous protocol [84]. Cyclin A (Santa-Cruz, Dallas, TX, USA) served as a control for cell fractionation.

### 4.7. Proliferation Assays

Cell viability and proliferation were measured using the Cell Proliferation Kit II (XTT) (Roche, New York, NY, USA). ATL cells were infected with shNotch shRNA or Puromycin-vector control for several days. Assays were performed in triplicate from multiple experiments. XTT readings were measured every day on an Anthos 2010 plate reader for up to 10 days.

### 4.8. GSI and GSI-Washout Assays

Jurkat, ATLT, ATL25, and MT1 cell lines were treated with or without GSI (Compound E, 1 μM) for 72 h. Cells were washed 3× in PBS and then treated with or without 40 μg/μL cycloheximide (CHX) for an additional 4 h. Cells were lysed in Trizol, and RNA was extracted for downstream applications. For ascertaining GSI-sensitivity (i.e., non-washout experiments), cells were treated with 1 μM GSI for 48 h (RNA) or 72 h (protein).

### 4.9. Chromatin Immunoprecipitation (ChIP)

ATL cells were grown in 10 cm dishes with and without 1 µM GSI for 72 h. Cells were cross-linked with formaldehyde for 10 min at 37 °C, followed by 1.25 M glycine for 10 min, and two cold PBS washes. Cell pellets were lysed in SDS lysis buffer (1% SDS, 10 mM EDTA, 50 mM Tris, pH 8.1) on ice, followed by sonication to cleave genomic DNA. Cell pellets were aliquoted and used for subsequent CHIP analysis using the ChIP Assay Kit (Upstate). For ChIP, lysates were diluted in CHIP dilution buffer (0.01% SDS, 1.1% Trion X-100, 1.2 mM EDTA, 16.7 mM Tris-HCl, pH 8.1, 167 mM NaCl), and 1% was kept toquantitate DNA for input. Salmon Sperm DNA/Protein A agarose-50% slurry (Upsate) was used to pre-clear lysates. Lysates were then incubated with anti-Notch (ab27526; Abcam) antibody overnight at 4 °C. Lysates were then washed in Low salt buffer, High salt buffer, LiCl buffer, and two TE Buffer washes (Upstate). Precipitates were eluted in Elution buffer (1% SDS, 0.1 M NaHCO_3_). Precipitates and input were incubated in 5 M NaCl to reverse cross-links. Proteinase K was used to remove protein. Extracts were then phenol/chloroform precipitated at −80 °C overnight. DNA was then spun down, washed in ethanol, and resuspended for downstream PCR applications. Hes-1 CHIP primers were as follows: F: CTGTGGGAAAGAAAGTTTGGG and R: GACCAAGGAGAGAGGTAGAC. Input and ChIP products were applied using SYBR green Real-Time PCR. The resulting PCR product was cloned into the pGEM-T vector and sequenced with the T7 primer. Sequencing was used to BLAST against the Hes-1 promoter sequence.

## Data Availability

Data sharing does not apply to this article as no datasets were generated or analyzed during the current study. Any plasmids generated can be obtained by contacting the corresponding author.

## Supplementary Materials

The following supporting information can be downloaded at: https://www.mdpi.com/article/10.3390/ijms25105130/s1.
