# Supplementary material for: Increased H19/miR-675 Expression in Adult T-Cell Leukemia Is Associated with a Unique Notch Signature Pathway
Source: Int J Mol Sci. 2024 May 8;25(10):5130. doi: 10.3390/ijms25105130 (PMC11120950; doi:10.3390/ijms25105130)
Supplement: Supplementary file 1 [file ijms-25-05130-s001.zip › ijms-2951880-supplementary.pdf]

Supplemental Material

Suppl S1: Notch and Maml expression in ATL and ALL cell lines.

Suppl S2: ALL Notch Gene Array expression and Hes-1, Hey-1, and c-Myc expression in ATL cells.

Suppl S3: ClusterGram Analysis and Gene Values for Notch Signaling Pathway Plus Array.

Suppl S4: Primers

Supple S5: Gene Values and Statistical Analysis.

Supplemental 1: Notch and Maml expression in ATL and ALL cell lines.

A.

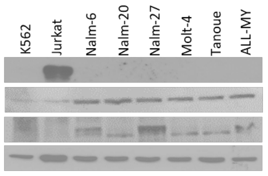

Suppl 1A: Western blot analysis of Maml-1, Maml-2, and NICD in ALL cell lines (Jurkat, Molt4, Tanoue, Nalm6, Nalm20, Nalm-27, and ALL-MY. Non-ALL, K562 served as control. Actin served as loading control.

B.

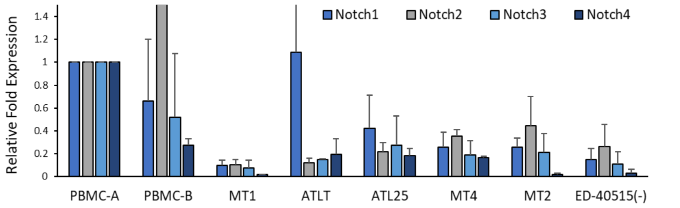

Suppl 1B: Real-time PCR analysis of Notch—1, 2,-3, and -4 in ATL cell lines (MT1, ATLT, ATL25, ED-40515(-)), HTLV infected cell lines (MT4 and MT2) and normal PBMCs. GAPDH served as an internal control. Results are from at least two distinct cDNA preparations and runs.

C.

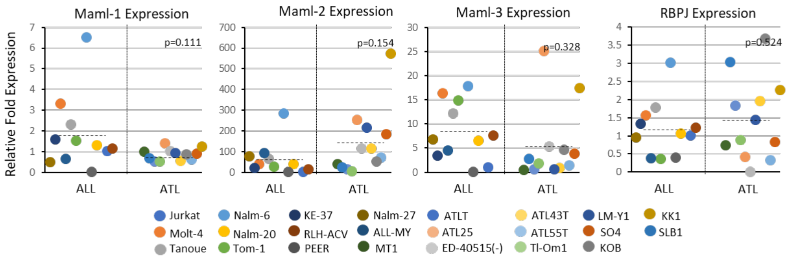

Suppl 1C: Real-time PCR analysis of Maml-1, -2, -3 and RBPJ expression in ATL cell lines (MT1, ATLT, ATL25, ED-40515(-), TI-Om1, ATL43T, ATL55T, SO4, KOB, KK1, SLB1 and LM-Y1) and ALL cell lines (Jurkat, Molt4, Tanoue, Nalm6, Nalm20, Tom-1, KE-37, RLH-ACV, PEER, Nalm-27, and ALL-MY). GAPDH served as a control. Results are from at least two distinct cDNA preparations and real-time PCR runs.

Supplemental 2: ALL Notch Gene Array expression and Hes-1, Hey-1, and c-Myc expression in ATL cells.

A.

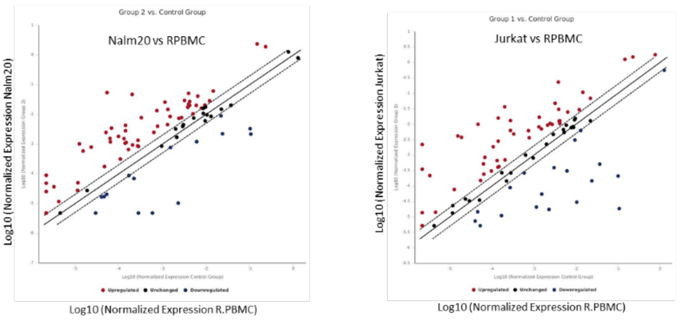

Suppl 2A: Graphs representing the Log10 normalized gene expression of Notch pathway genes of ALL cells (Nalm20 and Jurkat), compared to the Log10 normalized gene expression in PBMCs. Graphs are generated using Qiagen software.

B.

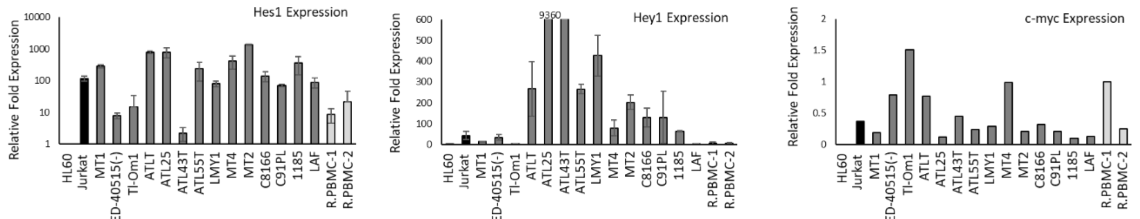

Suppl 2B: Real-time PCR analysis of Hes-1, Hey-1, and c-Myc expression in ATL cell lines (MT1, ATLT, ATL25, ED-40515(-), TI-Om1, ATL43T, ATL55T, and LM-Y1) and HTLV-I infected cells (MT4, MT2, C8166, C91PL, 1185, and LAF). Non-HTLV-I infected cells, HL60 and Jurkat served as controls, along with two resting PBMCs (rPBMCs). GAPDH served as a control. Results are from at least two distinct cDNA preparations and real-time PCR runs.

Supplemental 3: ClusterGram analysis of Human Notch Signaling Pathway Plus

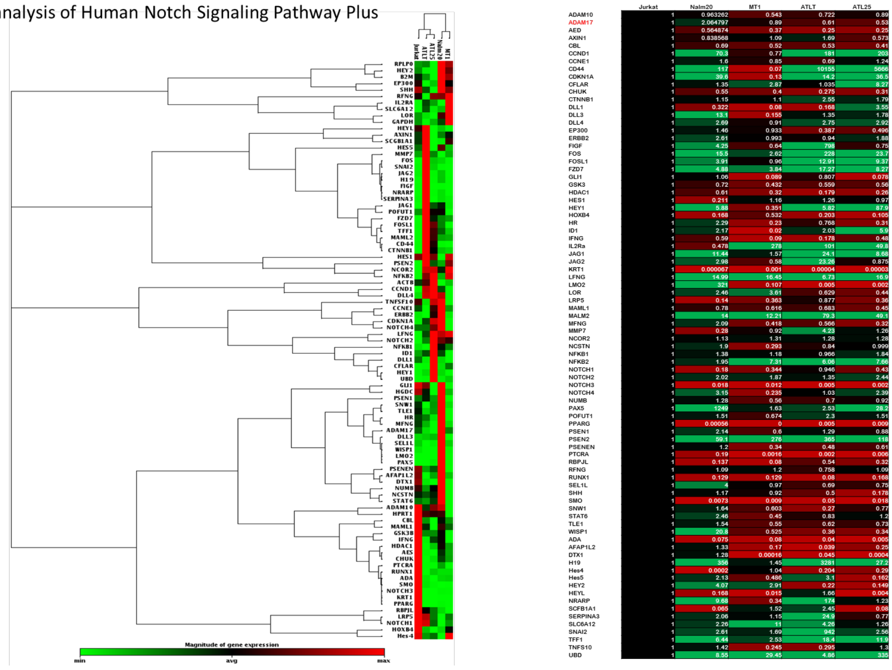

Supplemental 4: Primers

	Forward	Reverse
Notch-1	GCTGGCTGCAGGTTCTGCT	CCAGATGATGAGCTACCAGG
Notch-2	TTTGGCAACTAACGTAGAAACTCAAC	TGCCAAGAGCATGAATACAGAGA
Notch-3	GGTTCCCAGTGAGCACCCTTAC	GTGGATTCGGACCAGTCTGAGAG
Notch-4	CCCAGGAATCTGAGATGGAA	CCACAGCAAACTGCTGACAT
LFNG	TCGCTGTCAAGACCACCAAA	CCTCATCTTCCCCGTCAGTG
RFNG	CTTCCACTCTCACCTGGAGAACCTG	GTCTTGATGCAGGCTGAAGCCTCCAG
MFNG	CACCTGGAGACCCTGCAGCTGCTGAG	GAGCAGACAATGGAGGGAGCGAAATC
DTX1	CTACGATATGGACATCTGCATCAC	GTTCATCTGCGACATGCTGTTG
Hes-1	CTGGAAATGACAGTGAAGCACCT	ATTGATCTGGGTCATGCAGTTG
Hey-1	CCGAGATCCTGCAGATGACC	CCCGAAATCCCAAACTCCGA
JAG-1	ATCTGTGCTGCCTTTCAGTTT	GATCATGCCCGAGTGAGAA
c-Myc	CGTCTCCACACATCAGCACAA	TCTTGGCAGCAGGATAGTCCTT
HeyL	CGCAGAGGGATCATAGAGAAACG	GCCAGGGCTCGGGCATCAAAGAA
Hey-2	GAGTTGAGAAGACTTGTGCCAAC	GAGCGTGTGCGTCAAAGTAG
ID1	AAACGTGCTGCTCTACGACA	TCAGCGACACAAGATGCG
IL2Rα	CAGTGCGTCCAGGGATACAG	CTCTTCACCTGGAAACTGACTG
Maml-1	CACTCCCATGTCAGATGACTTGCAC	GGGTCTCACCCTGTGCTTACCA
Maml-2	GAGAATCTCTCTGCAGGACAAG	CAGAGTCTCTCCTGGCTCCTTC
Maml-3	AAGCCCAGGGACCGAGGCAA	GCAGCCTTGGAGGGGCTTGG
RBPJ-κ	GCTGACTTATGCATTGCCTCAGGA	CCACTGCTGTGAACTGGCATGAAA
CHUK	CATAGTTCTTCAGGATGTTGGTG	GTGGCTGTGTAAGGCTTATTCTC
PTCRA	CATCTGGTTCTCAGCCGGCAATG	GATGCCAGCTCCTCAGAAGGCAG
NFKB2	GATCGAGGTGGACCTGGTAACAC	GTCACATGCAGGACACCCAG
GIMAP5	CAAGAGCTGTACAAGAACATCG	GACCTCTTTCACCTTCCTGATG
ERBB2	CCTCTGACGTCCATCATCTC	ATCTTCTGCTGCCGTCGCTT
TFF1	GAGAACAAGGTGATCTGCGC	TGGTATTAGGATAGAAGCACC
SNAI2	TAGGAAGAGATCTGCCAGAC	CCCCAAGGCACATACTGTTA
miR-675	CCCAGGGTCTGGTGCGGAGA	CAGGGGCTGAGCGGTGAGGG
GAPDH	GTCGGAGTCAACGGATTTGGTCG	GGAACATGTAAACCATGTAGTTGAGG

	Forward	Reverse	Probe
H19	TGGTTGGAGTTGTGGAGACG	CACTTGCCAAGGTGGCTCA	CCTTGAGTCTCAGTACGAGTGTGCGTGAGT
Hes4	CAAGCCGGTCATGGAGAAG	TGGCGGGAGCTCTCTTT	CGAGCGCGTATTAACGAGAGCCTC
Hes5	GACTACAGCGAAGGCTACTC	CCGCTGGAAGTGGTACAG	CCAGCGACACGCAGATGAAGCT

Supplemental S5

	GSI<br>(Average Fold Change to DMSO)	Standard Deviation	p-value	Significance<br>p>0.05 *<br>p>0.01 **<br>p>0.001 ***
**Jurkat**				
**Hes1**	0.0255	0.0015	2.37E-06	***
**Hes4**	0.1285	0.0120208	9.511E-05	***
**Hes5**	0.32305	0.275135	0.073593	***
**Hey1**	0.234	0.036	0.002	**
**Hey2**	0.39	0.05	0.007	**
**HeyL**	0.53	0.07	0.011129	*
**ID1**	0.135	0.009	0.0001	
**Cmyc**	0.63	0.09	0.00073	***
**IL2Ra**	0.985	0.145	0.634364	
**DTX1**	0.123	0.003	0.192987	
**PTCRA**	0.275	0.025	0.034419	*
**NFKB2**	1.12	0.08	0.512565	
**GIMAP5**	0.22	0.04	0.0026	**
**LFNG**	0.795	0.045	0.045	*
**ERRB2**	1.005	0.0495	0.052046	
**TFF1**	0.92	0.0283	0.057191	
**SNAI2**	1.1	0.905	0.694005	
**JAG1**	0.264	0.186	0.0109	*
**Notch1**	0.7055	0.742	0.630989	
**MT1**				
**Hes1**	0.127	0.057	0.004	**
**Hes4**	0.554	0.145664	0.0494143	*
**Hes5**	0.82775	0.064842	0.064114	
**Hey1**	0.675	0.015	0.0021	**
**Hey2**	0.1455	0.0045	2.77E10-5	***
**HeyL**	0.6	0.099	0.003403	**
**ID1**	1.03	0.183848	0.689747	
**Cmyc**	0.455	0.025	0.00073	***
**IL2Ra**	1.29	0.06	0.634364	
**DTX1**	0.185	0.075	0.192987	
**PTCRA**	0.705	0.155	0.034419	*
**NFKB2**	1.14	0.12	0.512565	
**GIMAP5**	1.515	0.085	0.026	*
**LFNG**	0.94	0.03	0.184	
**ERRB2**	1.09	0.167	0.052046	
**TFF1**	0.82	0.1556	0.476707	
**SNAI2**	1.27	0.556	0.694005	
**JAG1**	0.659	0.46	0.0109	*
**Notch1**	0.929	0.286	0.296474	
**ATL25**				
**Hes1**	0.58	0.1058301	0.0023465	**
**Hes4**	0.534	0.1909188	0.0746497	
**Hes5**	0.36425	0.32633	0.110355	
**Hey1**	0.672667	0.1954	0.044051	*
**Hey2**	0.72	0.08556	0.03015	*
**HeyL**	4.505	0.289914	0.003403	**
**ID1**	1.06	0.183848	0.689747	
**Cmyc**	0.63	0.014142	0.00073	***
**IL2Ra**	1.075	0.1901919	0.634364	
**DTX1**	0.8135	0.136472	0.192987	
**PTCRA**	0.79	0.056569	0.034419	*
**NFKB2**	1.075	0.13435	0.512565	
**GIMAP5**	0.735	0.049497	0.017	*
**LFNG**	1.335	0.120208	0.058762	
**ERRB2**	1.035	0.021213	0.052046	
**TFF1**	1.031	0.083439	0.651729	
**SNAI2**	1.025	0.077782	0.694005	
**JAG1**	0.81	0.028284	0.0109	*
**Notch1**	0.965	0.035355	0.296474	
	PBMC<br>(Average Fold Change:<br>n=#PBMCs)	Primary ATL (Average Fold Change: n=#ATL)	p-value	Significance<br>p>0.05 *<br>p>0.01 **<br>p>0.001 ***
**Notch-1**	0.365157 (n=17)	0.523129 (n=23)	0.107186	
**Notch-2**	1.498373 (n=17)	0.854622 (n=23)	0.025352	*
**Notch-3**	0.482175 (n=17)	0.413342 (n=23)	0.595599	
**Notch-4**	0.363592 (n=17)	0.217121 (n=23)	0.304353	
**LFNG**	14.456 (n=18)	31.80118 (n=28)	0.00291	**
**RFNG**	1.914868 (n=18)	2.52651 (n=28)	0.038985	*
**MFNG**	1.477355 (n=18)	1.950028 (n=28)	0.102667	
**DTX1**	1.583477 (n=20)	7.755291 (n=22)	0.005918	**
**PSEN1**	3.671533 (n=18)	4.991018 (n=28)	0.154448	
**PSEN2**	1.478934 (n=18)	2.668966 (n=28)	0.05849	
**Maml1**	0.31800305 (n=20)	0.292432 (n=21)	0.767733	
**Maml2**	0.314237 (n=20)	0.383452 (n=21)	0.475818	
**Maml3**	0.491254 (n=20)	0.377991 (n=21)	0.191731	
**RBPJ-K**	1.356693 (n=18)	2.760729 (n=28)	0.016346	*
**Hes-1**	0.447342 (n=16)	0.161971 (n=28)	0.032	
**Hes-4**	1.231733 (n=18)	7.256128 (n=28)	0.140751	
**Hes-5**	0.46703 (n=18)	0.368926 (n=29)	0.501148	
**Hey-1**	6.481062 (n=16)	6.687755 (n=28)	0.899	
**Hey-2**	0.98041 (n=16)	1.08688936 (n=28)	0.855	
**Hey-L**	3.571415 (n=18)	8.363886 (n=28)	0.184641	
**c-Myc**	2.045568 (n=16)	4.295062 (n=28)	0.005	**
**ID1**	0.4861 (n=16)	0.099377 (n=28)	0.077	
**miR-675**	2.761015 (n=18)	6.121786 (n=29)	0.014947	*
**H19**	1.675244602 (n=17)	31.35084 (n=51)	0.007	**
